# Supplementary figures and images for: Deciphering the RNA-based regulation mechanism of the phage-encoded AbiF system in Clostridioides difficile
Source: PLoS Genet. 2025 Aug 19;21(8):e1011831. doi: 10.1371/journal.pgen.1011831 (PMC12373285; doi:10.1371/journal.pgen.1011831)

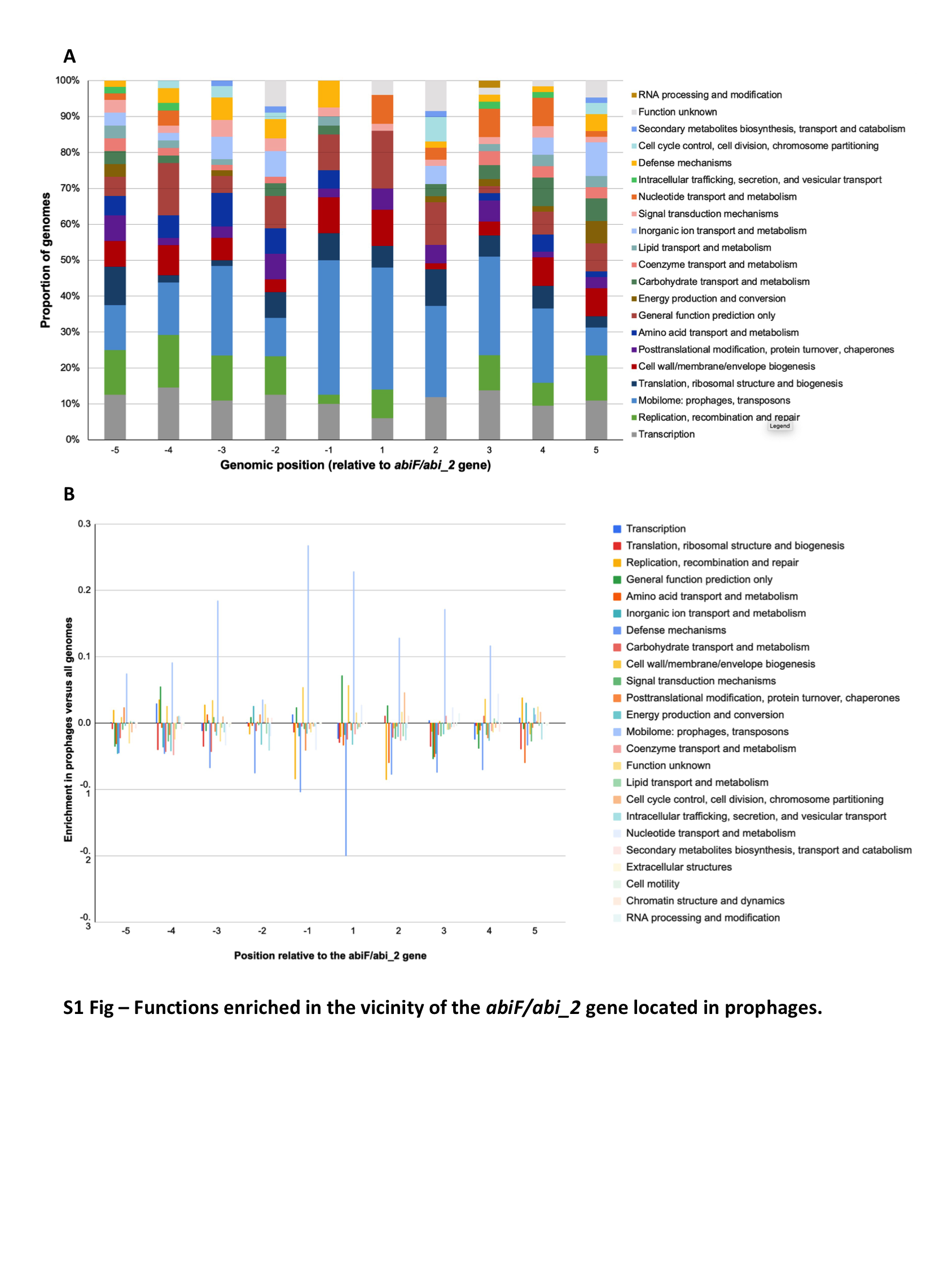

Supplement: S1 Fig — (A) Functions at the -5 to +5 positions surrounding abiF/abi_2 located in prophages (N = 288 genomes). (B) Relative proportion of functions in prophages vs 2,312 genomes where abiF/abi_2 is not located in a prophage. (S1_Fig.TIFF) [file pgen.1011831.s001.tiff]

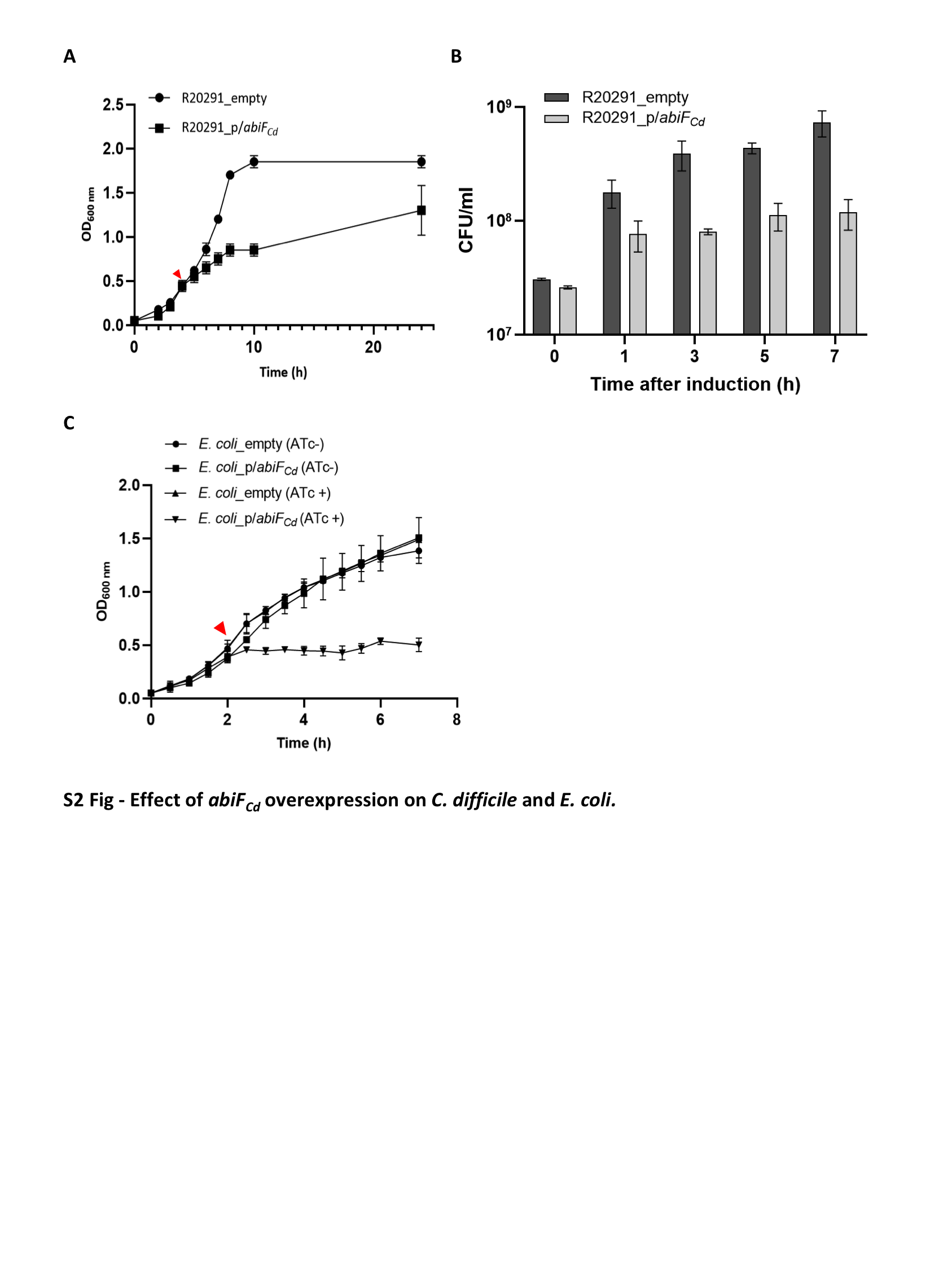

Supplement: S2 Fig — (A) Growth curve and (B) CFU/ml of C. difficile R20291 carrying p/abiFCd or empty plasmid (empty) in TY supplemented with Tm and with (ATc +) or without induction. Induction of the Ptet promoter by 250ng/mL ATc is indicated by the red arrow. (C) Growth curve of E. coli carrying p/abiFCd or empty plasmid (empty) in LB supplemented with Cm and 250ng/mL ATc (indicated by a red arrow). Plotted values represent the mean standard error of the mean (N = 3 biologically independent samples). (S2_Fig.TIFF) [file pgen.1011831.s002.tiff]

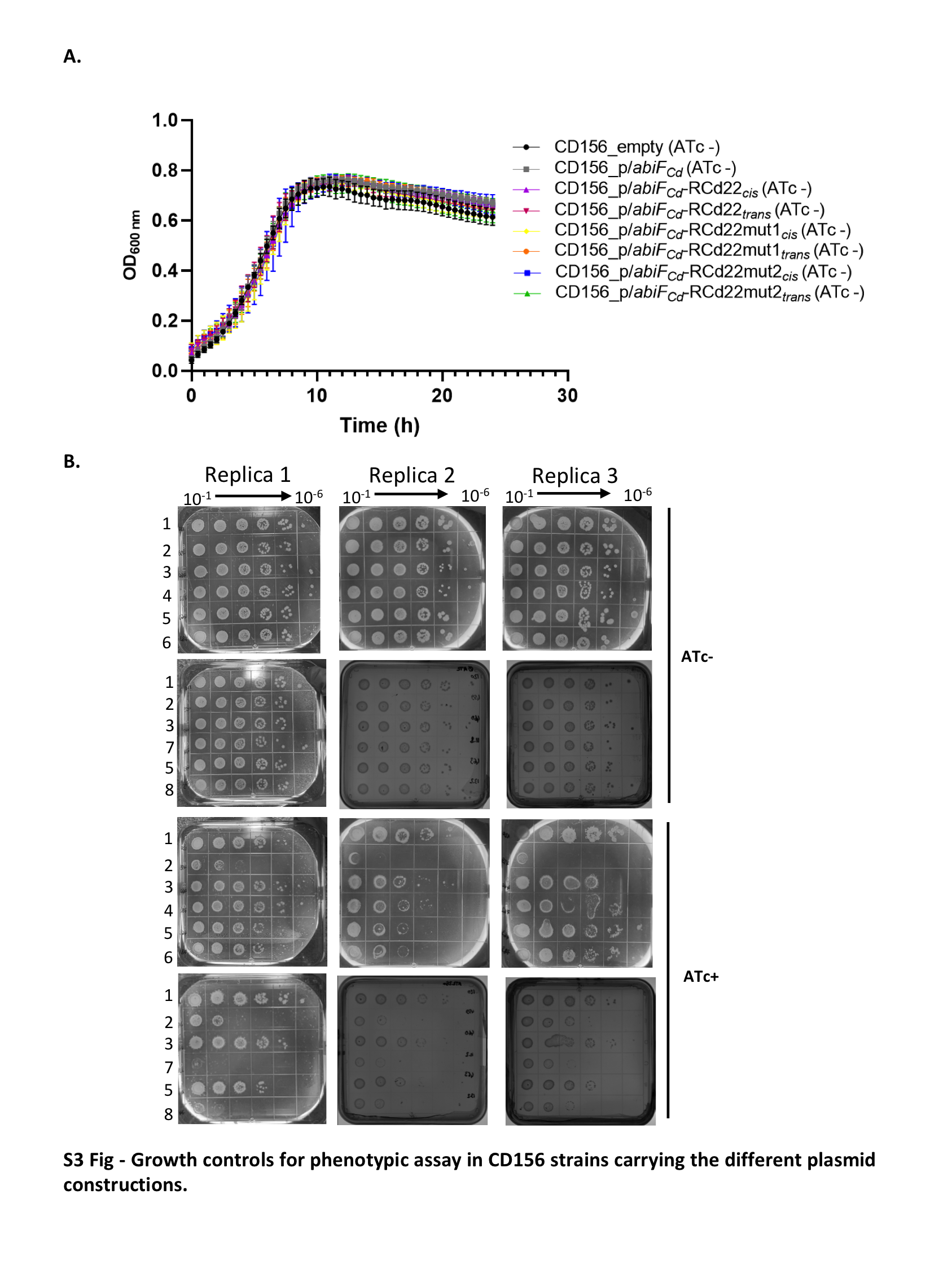

Supplement: S3 Fig — (A) C. difficile CD156 strains carrying empty plasmid (empty), overexpressing abiFCd (p/abiFCd), or co-expressing abiFCd and RCd22 (in cis or in trans, with or without mutations 1 or 2) were grown in TY supplemented with Tm without induction (ATc -). (B) Biological triplicate of spot assay of CD156 strains carrying empty plasmid (1), overexpressing abiFCd (2), co-expressing abiFCd and RCd22 in cis (3), with mutation 1 (4) or mutation 2 (7) or in trans (5) with mutation 1 (6) or mutation 2 (8). Spot assay is realized on TY plate supplemented with Tm and with or without inducer (ATc + or -). (S3_Fig.TIFF) [file pgen.1011831.s003.tiff]

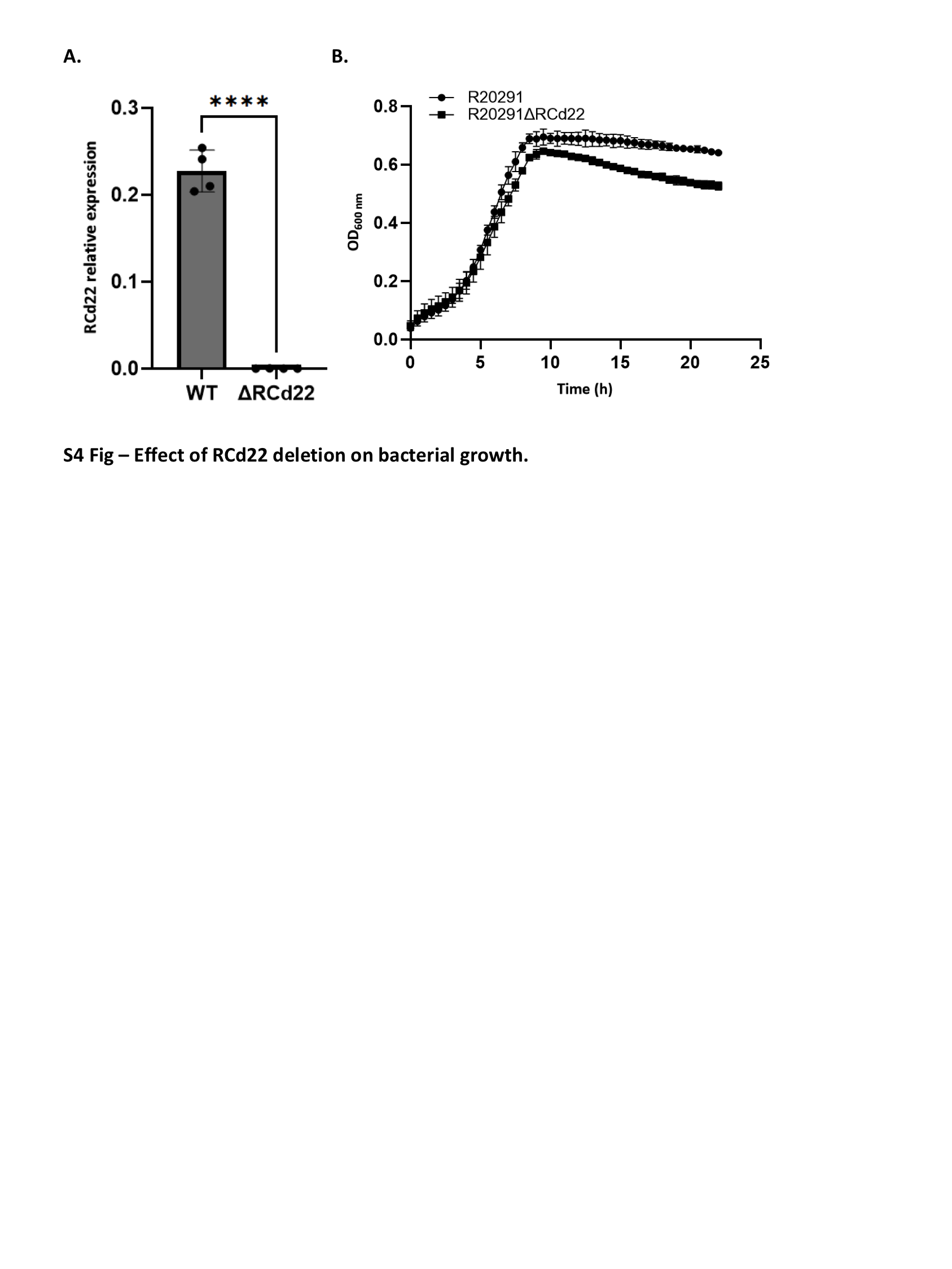

Supplement: S4 Fig — (A) Relative expression of RCd22 to the reference 16S RNA gene in C. difficile R20291 and ΔRCd22 strains. (B) Growth curve of C. difficile R20291 strain in TY. Plotted values represent the mean standard error of the mean (N = 3 biologically independent samples). (S4_Fig.TIFF) [file pgen.1011831.s004.tiff]

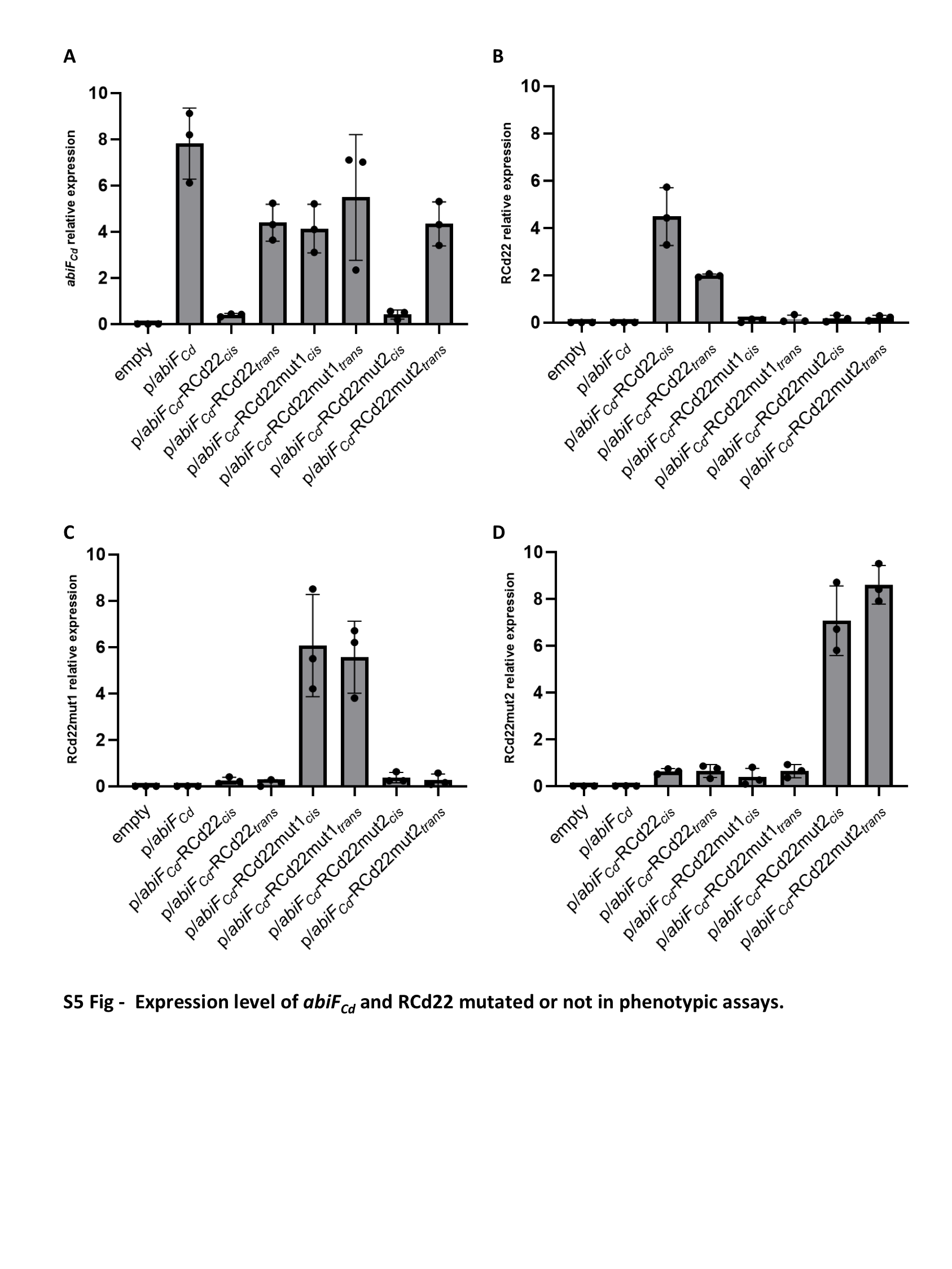

Supplement: S5 Fig — For each strain carrying the different plasmids, the expression of abiFCd (A), RCd22 (B), RCd22mut1 (C) and RCd22mut2 (D) were controlled by RT-qPCR. Total mRNAs were collected after 2h of induction with ATc, during phenotypic assay. The level of expression is relative to the reference DNApol III gene. (S5_Fig.TIFF) [file pgen.1011831.s005.tiff]

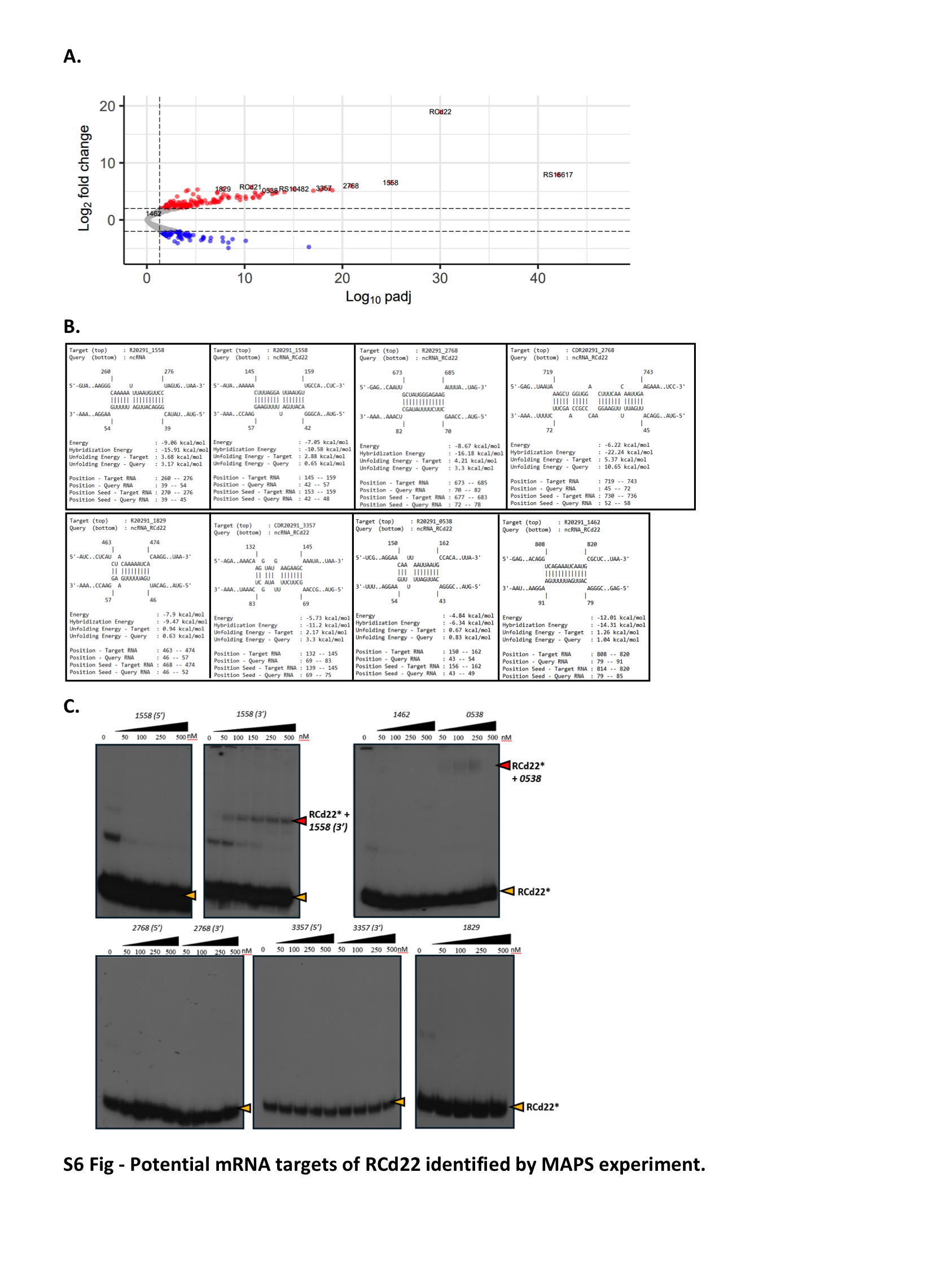

Supplement: S6 Fig — (A) Volcano plot of mRNA targeted by RCd22 after MAPS experiment. Red dots indicate enriched RNA targeted by RCd22 compared to the control (MS2tag alone). (B) Hybridization energy of RCd22 with five candidate target sequences calculated with IntaRNA. Sequence of mRNA targets and RCd22 started from predicted transcript starting site to predicted terminator. In case of operon, the sequence started from the intergenic region of the target gene. (C) Shift assay performed with radioactive RCd22 (RCd22*) transcribed in vitro and mixed with different concentrations of potential mRNA targets identified by MAPS and transcribed in vitro. mRNA of abiFCd (R20291_1462) was also tested. (S6_Fig.TIFF) [file pgen.1011831.s006.tiff]

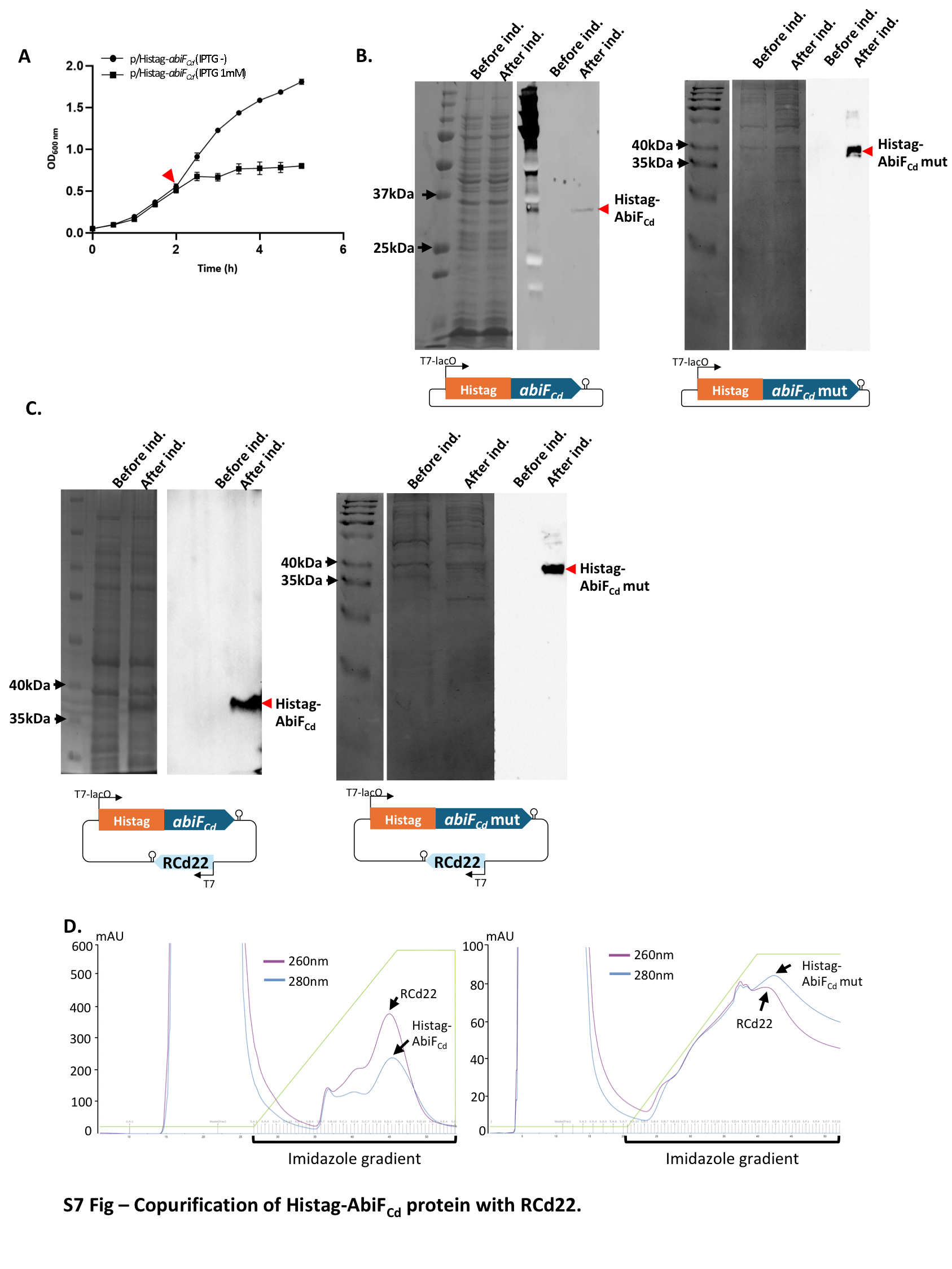

Supplement: S7 Fig — (A) Verification of Histag-abiFCd effect on growth of E. coli BL21(DE3) in LB supplemented with Cm, and with or without induction (1mM IPTG, indicated by a red arrow). Plotted values represent the mean standard error of the mean (N = 3 biologically independent samples). (B) Coomassie and Western Blot (HRP anti-Histag antibody) of total protein extract from E. coli BL21(DE3) carrying the p343 plasmid (Histag-abiFCd) or the p438 plasmid (Histag-abiFCd carrying mutations R202D and H207D) before and after 4h of induction with 1mM IPTG. (C) Coomassie and Western Blot (HRP anti-Histag antibody) of total protein extract from E. coli BL21(DE3) carrying the p353 plasmid (Histag-abiFCd and RCd22trans) or the p439 plasmid (Histag-abiFCd carrying mutations R202D and H207D, and RCd22trans) before and after 4h of induction with 1mM IPTG. (D) Graph of Histag-AbiFCd or Histag-AbiFCd carrying mutations R202D and H207D (Histag-AbiFCdmut) copurification during HisTrap affinity column, obtained by AKTA FPLC system. (S7_Fig.TIFF) [file pgen.1011831.s007.tiff]

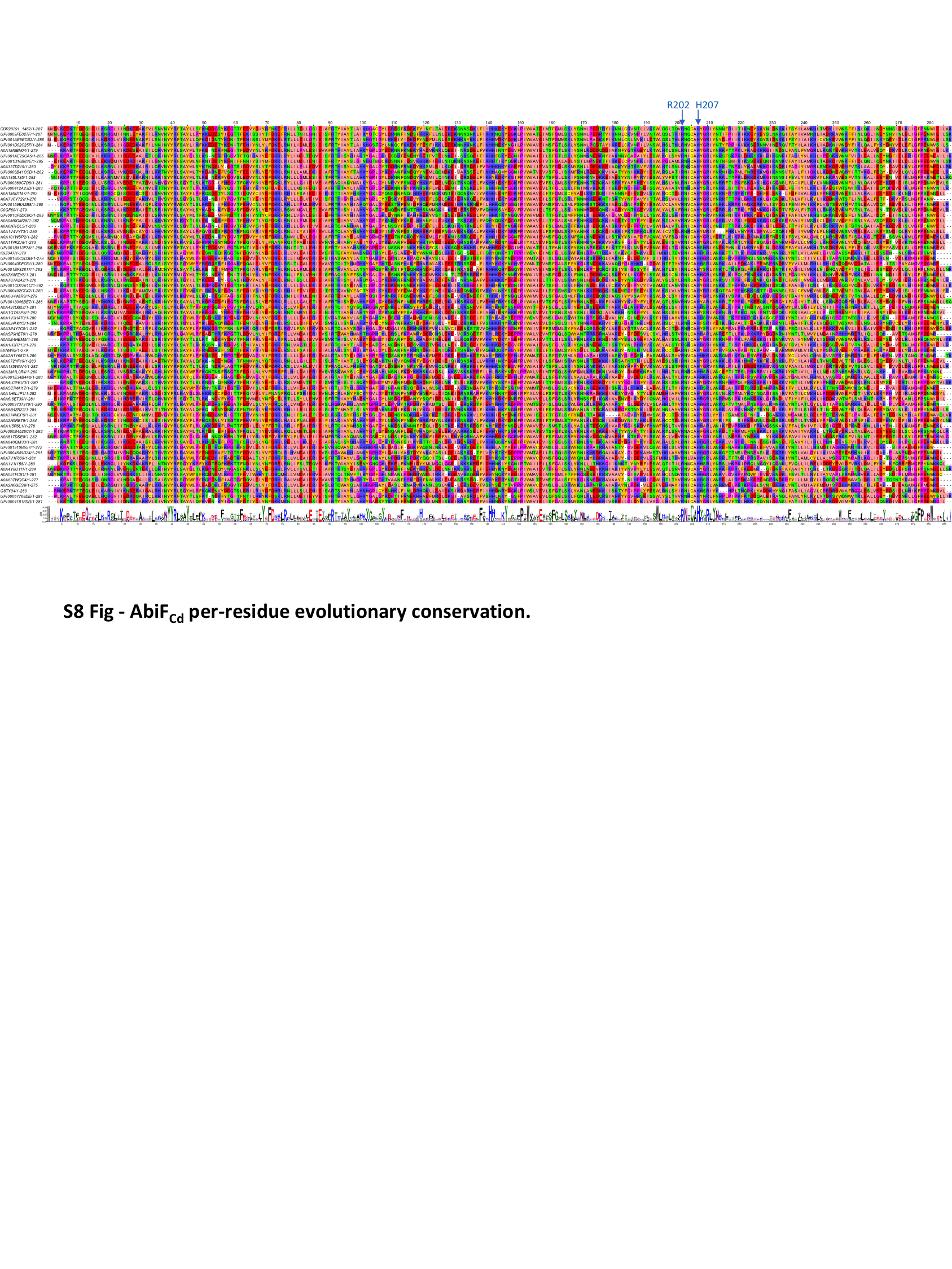

Supplement: S8 Fig — Upper panel: filtered AbiFCd multiple sequence alignment obtained with MMseqs2. The top 100 sequences (see Methods) were further filtered to remove redundancy at 70% sequence identity for easier visualization, and displayed using the Zappo color code with Jalview [75]. Lower panel: representation of this multiple sequence alignment as a logo, using WebLogo [76]. Positions R202 and H207, mutated in the present work, are highlighted with blue arrows. Sequence identifiers are obtained from the UniRef30 database version 2202 [73]. (S8_Fig.TIFF) [file pgen.1011831.s008.tiff]

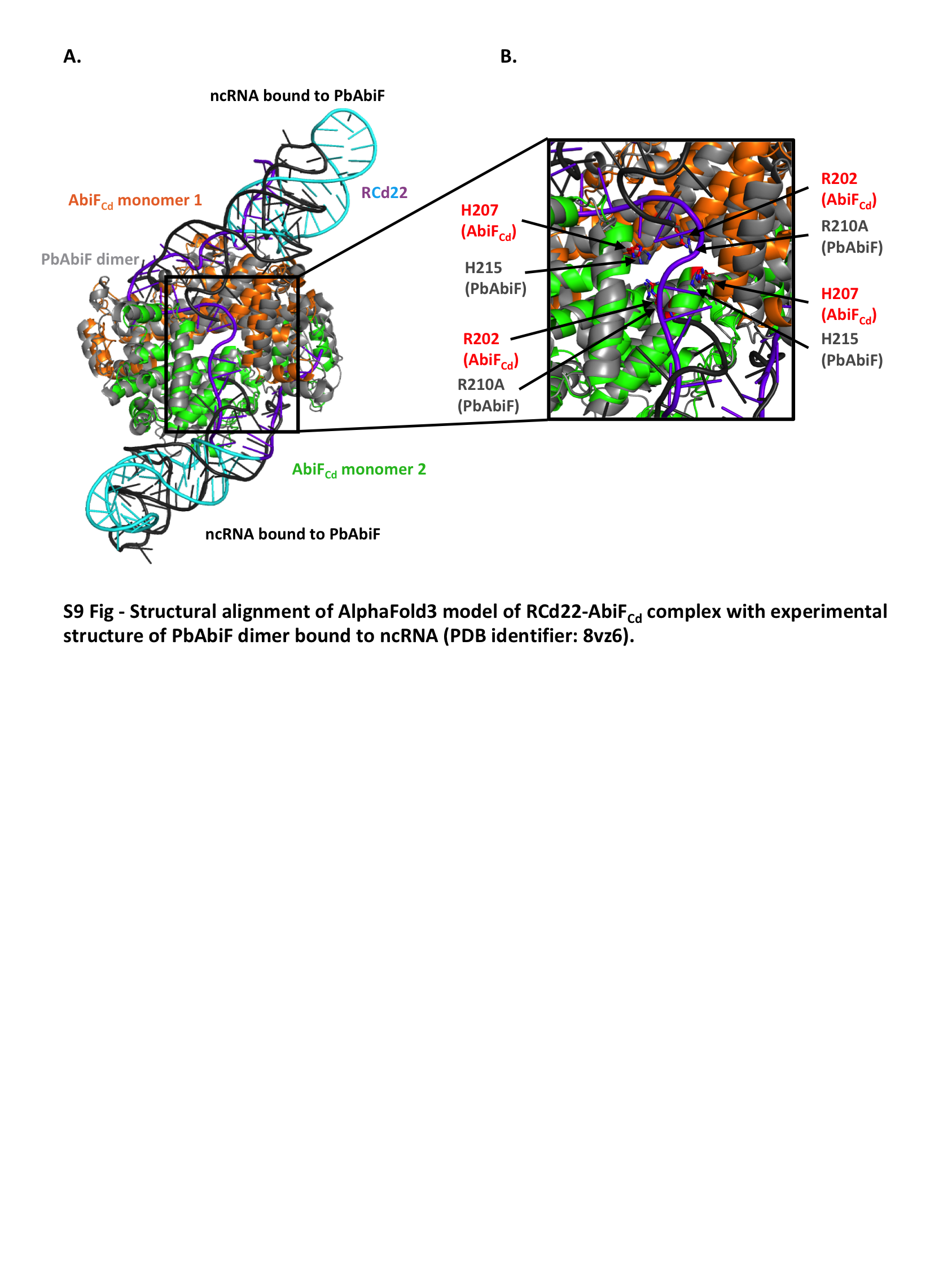

Supplement: S9 Fig — The AlphaFold3 model, colored as in Fig 8B and 8C (AbiFCd in orange and green, RCd22 in purple with abiF motifs in cyan), was structurally aligned with the experimental structure of the PbAbiF dimer (in light grey) bound to two copies of a ncRNA (in dark grey). (A) Overall view. (B) Focus on the region of amino acids R202 and H207 in AbiFCd, structurally aligned to R210 and H215 in PbAbiF. (S9_Fig.TIFF) [file pgen.1011831.s009.tiff]
